# The chicken gene nomenclature committee report

**DOI:** 10.1186/1471-2164-10-S2-S5

**Published:** 2009-07-14

**Authors:** David W Burt, Wilfrid Carrë, Mark Fell, Andy S Law, Parker B Antin, Donna R Maglott, Janet A Weber, Carl J Schmidt, Shane C Burgess, Fiona M McCarthy

**Affiliations:** 1 Department of Genomics and Genetics, Roslin Institute and Royal (Dick) School of Veterinary Studies, Midlothian EH25 9PS, UK; 2 Department of Cell Biology and Anatomy, Medical Research Building, 1656 E. Mabel Street, P.O. Box 245217, Tucson, AZ 85724, USA; 3 National Center for Biotechnology Information, National Library of Medicine, 6800 Rockville Pike, Bethesda, MD 20894, USA; 4 Department of Animal and Food Sciences, University of Delaware, Newark, DE 19706, USA; 5 Department of Basic Sciences, College of Veterinary Medicine, Mississippi State University, Mississippi State, MS 39762, USA; 6 Mississippi State University Institute for Digital Biology, Mississippi State University, Mississippi State, MS 39762, USA; 7 Mississippi Agriculture and Forestry Experiment Station, Mississippi State University, Mississippi State, MS 39762, USA; 8 MSU Life Sciences and Biotechnology Institute, Mississippi State University, Mississippi State, MS 39762, USA

## Abstract

Comparative genomics is an essential component of the post-genomic era. The chicken genome is the first avian genome to be sequenced and it will serve as a model for other avian species. Moreover, due to its unique evolutionary niche, the chicken genome can be used to understand evolution of functional elements and gene regulation in mammalian species. However comparative biology both within avian species and within amniotes is hampered due to the difficulty of recognising functional orthologs. This problem is compounded as different databases and sequence repositories proliferate and the names they assign to functional elements proliferate along with them. Currently, genes can be published under more than one name and one name sometimes refers to unrelated genes. Standardized gene nomenclature is necessary to facilitate communication between scientists and genomic resources. Moreover, it is important that this nomenclature be based on existing nomenclature efforts where possible to truly facilitate studies between different species. We report here the formation of the Chicken Gene Nomenclature Committee (CGNC), an international and centralized effort to provide standardized nomenclature for chicken genes. The CGNC works in conjunction with public resources such as NCBI and Ensembl and in consultation with existing nomenclature committees for human and mouse. The CGNC will develop standardized nomenclature in consultation with the research community and relies on the support of the research community to ensure that the nomenclature facilitates comparative and genomic studies.

## Background

Chicken is the foremost non-mammalian vertebrate biomedical model organism and it is a principal biomedical model for understanding basic biology, behaviour and disease. As the *de facto *model bird genome, chicken also occupies a unique and important evolutionary niche and chicken is often used in comparative and evolutionary genomics. Comparative research using chicken has made seminal contributions to understanding infectious disease, cancer, cell biology, embryology, gene regulation, immunology, and nutrition. However, lack of standardized gene nomenclature prevents researchers from exploiting the full potential of the chicken for comparative and functional genomic studies. Currently, chicken genes are published under more than one name and one name sometimes refers to unrelated genes. Moreover, a large number of chicken genes were predicted during the final stages of the chicken genomic sequence assembly based on sequence similarity to known (mammalian) genes, chicken ESTs and *de novo *prediction [1]. Updates of gene models and gene prediction pipelines from different sources have compounded this problem. Standardized nomenclature will facilitate communication between scientists and enable comparative biology studies.

The HUGO Gene Nomenclature Committee (HGNC) approves a unique short-form abbreviation (gene symbol) and a longer descriptive name for human genes [2]. Gene symbols are unique Latin letters and Arabic numerals (<7 characters) that facilitate computation. The longer descriptive gene name aims to convey the character or function of the gene and yet be concise. Assigning names and the unique short abbreviations necessitates corresponding with authors, reading the literature and performing data analyses. When possible the symbol used in publications is retained but if the symbol has already been used for another gene or if the gene is a member of a gene family an alternative symbol is approved. Interspecies nomenclature confusion is avoided by assigning the same gene symbol to orthologous genes.

Since chicken is the model avian genome, determining core orthologs that exist between avian and mammalian species is particularly informative [1,3-6]. The utility of standardized orthologous gene names is one of the strongest arguments for approved nomenclature and cooperation between the nomenclature committees of different species. Analyzing comparative maps without standardized gene nomenclature is difficult. Despite a proposal for a standardized chicken gene nomenclature in 1995 [7], it wasn't until the 2007 Chicken Development Meeting (April, Barcelona, Spain) that a Chicken Gene Nomenclature and Annotation Workshop was convened to create a pipeline for standardizing chicken nomenclature. A Chicken Gene Nomenclature Committee (CGNC) had been formed several years earlier and NCBI recognizes the CGNC as the official chicken gene naming entity. At the 2008 Avian Genome Meeting, Ensembl representatives also adopted HGNC approved nomenclature for genes with direct human orthologs and for which Ensembl and NCBI concur regarding their identity.

## Nomenclature guidelines

The chicken research community formally embraced standardized gene nomenclature more than a decade ago [7]. In keeping with HGNC guidelines, chicken genes will be assigned a unique gene symbol and gene name in consultation with researchers and in concordance with assigned human gene nomenclature, where such nomenclature exists for human:chicken ortholog pairs. The CGNC will work closely with existing vertebrate gene nomenclature committees and both HGNC and Mouse Gene Nomenclature Committee (MGNC) representatives serve as advisors on the CGNC. Current HGNC guidelines http://www.genenames.org/guidelines.html state that gene names should be brief and specific and should convey the character or function of the gene, the first letter of the symbol should be the same as that of the name in order to facilitate alphabetical listing and grouping, gene names should follow American spelling and tissue specificity and molecular weight designations should be avoided. Gene symbols must be unique, be representative of the descriptive gene name, should contain only Latin letters and Arabic numerals, should not contain punctuation, should not contain "G" for gene, and should not contain any reference to species (eg. "c" or "ch" for chicken).

The CGNC database aims to capture aliases or synonyms for chicken genes. In many cases where a standardized gene name is applied to a chicken gene there will be other names used to report this gene – often based on separate reports of the gene in published literature. By making this data available, researchers will be able to better find and evaluate available literature for the gene(s) they are studying.

Traditionally, where human genes names are based on orthology to other species, the name of the originating species is included in the gene name. However, due to the increasing number of sequenced species and use of orthologs to assign nomenclature, CGNC will not denote species in assigning gene names. For example the human gene name for HGNC:30387 is vitelline membrane outer layer 1 homolog (chicken) and in chicken this will become vitelline membrane outer layer 1.

## Assigning gene nomenclature

### Nomenclature based on human orthologs

Since avian gene nomenclature is to be based on existing human gene nomenclature where possible [8], the first step is to identify strict 1:1 chicken:human orthologs and assign these chicken genes symbols and names based upon the human nomenclature. Predicted chicken:human orthologs are now available from several tools and resources, including Ensembl, Evola(H-InvDB), HomoloGene, Inparanoid, OMA and Treefam [9-14]. The HGNC Comparison of Orthology Predictions (HCOP) tool allows users to view the ortholog predictions for each of these tools together with human gene nomenclature information [15]. Initial efforts to provide information about genes predicted during the chicken genome sequencing effort used orthology prediction tools to assign standardized nomenclature based upon human gene nomenclature for 6,012 chicken genes [16].

A chicken gene annotation tool (GENENAMES) has been created http://genenames.roslin.ac.uk/ and approximately 8,200 gene names with a confirmed 1:1 orthology to human have been approved by the CGNC. This data is based upon a total of 29, 071 chicken genes from Ensembl (Release 48) and Entrez Gene. For example, in Ensembl (Release 48) 6,743 of these chicken genes have a clear 1:1 orthology with a human gene that has been assigned HGNC nomenclature. These orthologs are initially identified using bioinformatics and are then confirmed manually by editors of the chicken GENENAMES database. GENENAMES editors are typically members of the chicken community (Table 1) who have interest/expertise in particular genes. For more information about GENENAMES editors please contact the CGNC genenames@roslin.ed.ac.uk.

**Table 1:** Current editors of chicken GENENAMES database. The current editors of the chicken GENENAMES database and their affiliations are shown. Researchers interested in assisting with chicken gene nomenclatures may contact the CGNC genenames@roslin.ed.ac.uk.

*CGNC Editor*	*Affiliation*
Parker Antin	UA
Elspeth Brufurd	HGNC
Dave Burt	RI
Wilfrid Carrë	RI
Mark Fell	RI
Andy Law	RI
Fiona McCarthy	MSU
Dennis Prickett	BBSRC
Jacqueline Smith	BBSRC
Michael Watson	BBSRC
Janet Weber	NCBI
John Young	BBSRC

UA – University of Arizona; HGNC – HUGO Gene Nomenclature Committee; RI – Roslin Institute and Royal (Dick) School of Veterinary Studies; MSU – Mississippi State University; BBSRC – Biotechnology and Biological Sciences Research Council.

Nomenclature provided by CGNC is used by NCBI Entrez Gene and will be distributed via NCBI to the Ensembl *Gallus gallus *browser. Importantly, these genes have also been assigned a universal CGNC gene ID to reliably link chicken genes across all databases. However, maintaining standard nomenclature at public databases is a continuous process. For example, NCBI's Entrez Gene and RefSeq resources use the nomenclature from CGNC when it is available. If the CGNC nomenclature is not yet released, NCBI assigns the symbol and full name from the human orthologs named by HGNC and identified by HomoloGene, according to the rules defined in this paper. Symbols provided by authors of publications or submitters of gene-specific sequences are retained as alternates.

In cases where the human ortholog is identified by its chromosomal location, HGNC guidelines recommend the practice adopted by MGNC: that is, when a predicted human gene is designated by the chromosome of origin, the letters "orf" for open reading frame and a number (C#orf#), we will prefix the human symbol with the chicken chromosome number. For example, the chicken ortholog for human C1orf26 (HGNC:16785) is located on chromosome 8 and is designated C8H1orf26 "chromosome 8 open reading frame, human C1orf26". These names will be replaced by more informative nomenclature as more becomes known about these genes and their function.

### Nomenclature for novel chicken genes

Novel chicken genes fall into two broad categories: novel genes predicted by bioinformatics gene prediction programs and novel chicken genes that have been studied prior to the completion of chicken genome sequencing. Putative open reading frames from the NCBI gene prediction pipeline are designated with a locus number, for example LOC777587 while the novel Ensembl genes not predicted by the NCBI pipeline are assigned Ensembl identifiers. In cases where there is no strict 1:1 human ortholog that has been assigned nomenclature, the LOC# or Ensembl ID will be used as the temporary gene symbol.

Chicken genes that that do not have strict 1:1 human orthologs will be manually curated and assigned nomenclature on the basis of their current names. Only unique symbols and gene names will be approved. Where individual researchers have named these genes, they will be asked to provide feedback on nomenclature within current nomenclature guidelines retaining a name as close to the original name as possible. Where more than one name for a gene exists because more than one author has published a name for the gene, the author with the first publication will have precedence. The exception to this rule is if one name has gained common acceptance within the research community. Dr. Elspeth Bruford is the HGNC representative on the CGNC and her advice and experience will be sought as required.

### Nomenclature for gene families

An exception to the rule of preferring feedback from publishing authors is the case of gene families. Hierarchical symbols for both structural and functional gene families will be used where possible because a stem (or root) symbol as a basis for a symbol series allows easy identification of other family members in both database searches and the literature. Examples of gene families include the G protein-coupled receptor genes (GPR1, GPR2, GPR3, etc) and the cytochrome P450 superfamily (CYP1A1, CYP21A2, CYP51A1, etc). In this case, consecutive symbols take precedence over those published, but again this will be a consultative matter between the CGNC and the research community.

We expect that in the case of gene families, specialized knowledge will be required to correctly determine members of gene families, their order and nomenclature. For example, considerable work has already been done on providing nomenclature for the chicken major histocompatibility B complex genes [17-21]. We expect to utilize the work done by experts in this field. Moreover, CGNC will follow HGNC policy of convening specialized working groups from the community to ensure that gene nomenclature meets community requirements.

## Future directions

The chicken genome has become the "foundation reference genome" for assembly and annotation of genome sequences for all archosaurs. Both turkey and zebra finch are undergoing genome sequencing (turkey because of its importance as an agricultural species and as a biomedical model for aging, while zebra finch is a biomedical model for behaviour and vocalization). Genome sequencing of these avian species utilizes chicken genome information to facilitate assembly and annotation. EST projects are already underway for additional bird species (eg. quail and condor) and in the three to five year timeframe, it is likely that sequencing of additional bird genomes will be undertaken. Moreover, comparative genetics studies in other archosaur species, including crocodile and alligator, are leveraging chicken gene information. Chicken's importance as a reference genome for these non-mammalian species underlines the fundamental importance of chicken in increasing numbers comparative genomics studies. Standardized gene nomenclature for chicken serves as a stepping off point for many other non-mammalian species.

Although work to provide standardized nomenclature for chicken genes is ongoing, the CGNC relies on the support of the research community. Only with community input and support will gene nomenclature be relevant for community needs, facilitate comparative biology and promote data exchange among both researchers and public resources. Researchers may submit comments, erratum and suggestions or requests for gene names to genenames@roslin.ed.ac.uk. The CGNC will publish regular reports of progress and calls for working groups to study gene families at avian conferences and using avian newsgroups.

## Competing interests

The authors declare that they have no competing interests.

## Authors' contributions

DWB and FMM contributed equally to the writing of the first draft; all other authors contributed writing to the manuscript and were involved in the editorial process. WC, MF, AL and DB created and implemented the GENENAMES database. PBA, CJS, JAW and FMM have all manually curated genes in the GENENAMES database. JAW and DRM implemented functions to assign names to novel Entrez gene records according to these standards, and enabled the CGNC nomenclature to be linked to NCBI Entrez Gene entries and NCBI Reference sequences (RefSeqs). SCB, PBA and FMM devised the mechanisms for assigning nomenclature for different types of chicken genes.
